# Supplementary material for: HIVprotI: an integrated web based platform for prediction and design of HIV proteins inhibitors
Source: J Cheminform. 2018 Mar 9;10:12. doi: 10.1186/s13321-018-0266-y (PMC5845081; doi:10.1186/s13321-018-0266-y)
Supplement: Supplementary file 2 — Additional file 2. Source code of HIVProtI web server. [file 13321_2018_266_MOESM2_ESM.zip › HIVprotI_Source-code/hivprotI/weka-3-6-6/doc/weka/clusterers/forOPTICSAndDBScan/Utils/EpsilonRange_ListElement.html]

EpsilonRange\_ListElement


---


|  |  |  |
| --- | --- | --- |
| **Overview** \| **Package** \| **Class** \| **Tree** \| **Deprecated** \| **Index** \| **Help** \| **Weka's home** | |  |
| PREV CLASS   **NEXT CLASS** | **FRAMES**    **NO FRAMES**     **All Classes** |  |
| SUMMARY: NESTED \| FIELD \| CONSTR \| METHOD | DETAIL: FIELD \| CONSTR \| METHOD |  |


---


## weka.clusterers.forOPTICSAndDBScan.Utils Class EpsilonRange\_ListElement

```
java.lang.Object
  weka.clusterers.forOPTICSAndDBScan.Utils.EpsilonRange_ListElement
```

**All Implemented Interfaces:**: RevisionHandler

---

``` public class EpsilonRange_ListElement extends java.lang.Object implements RevisionHandler ```

EpsilonRange\_ListElement.java

**Version:**
:   $Revision: 1.3 $

**Author:**
:   Matthias Schubert (schubert@dbs.ifi.lmu.de), Zhanna Melnikova-Albrecht (melnikov@cip.ifi.lmu.de), Rainer Holzmann (holzmann@cip.ifi.lmu.de)

---

**Constructor Summary**

`EpsilonRange_ListElement(double distance,
DataObject dataObject)`
  
          Constructs a new Element that is stored in the ArrayList which is
built in the k\_nextNeighbourQuery-method from a specified database.


| **Method Summary** | |
| --- | --- |
| `DataObject` | `getDataObject()`             Returns this dataObject |
| `double` | `getDistance()`             Returns the distance that was calulcated for this dataObject (The distance between this dataObject and the dataObject for which an epsilon-range-query was performed.) |
| `java.lang.String` | `getRevision()`             Returns the revision string. |

**Methods inherited from class java.lang.Object**

`equals, getClass, hashCode, notify, notifyAll, toString, wait, wait, wait`

**Constructor Detail**

### EpsilonRange\_ListElement

```
public EpsilonRange_ListElement(double distance,
                                DataObject dataObject)
```

:   Constructs a new Element that is stored in the ArrayList which is
    built in the k\_nextNeighbourQuery-method from a specified database.
    This structure is chosen to deliver not only the DataObjects that
    are within the epsilon-range but also deliver the distances that
    were calculated. This reduces the amount of distance-calculations
    within some data-mining-algorithms.

    **Parameters:**: `distance` - The calculated distance for this dataObject: `dataObject` - A dataObject that is within the epsilon-range


**Method Detail**

### getDistance

```
public double getDistance()
```

:   Returns the distance that was calulcated for this dataObject
    (The distance between this dataObject and the dataObject for which an epsilon-range-query
    was performed.)

    :   **Returns:**: distance

---


### getDataObject

```
public DataObject getDataObject()
```

:   Returns this dataObject

    :   **Returns:**: dataObject

---


### getRevision

```
public java.lang.String getRevision()
```

:   Returns the revision string.

    :   **Specified by:**: `getRevision` in interface `RevisionHandler`
    :   **Returns:**: the revision


---


|  |  |  |
| --- | --- | --- |
| **Overview** \| **Package** \| **Class** \| **Tree** \| **Deprecated** \| **Index** \| **Help** \| **Weka's home** | |  |
| PREV CLASS   **NEXT CLASS** | **FRAMES**    **NO FRAMES**     **All Classes** |  |
| SUMMARY: NESTED \| FIELD \| CONSTR \| METHOD | DETAIL: FIELD \| CONSTR \| METHOD |  |


---
